# Role of Gut Microbiota through Gut–Brain Axis in Epileptogenesis: A Systematic Review of Human and Veterinary Medicine

**DOI:** 10.3390/biology11091290

**Published:** 2022-08-30

**Authors:** Floriana Gernone, Annamaria Uva, Marco Silvestrino, Maria Alfonsa Cavalera, Andrea Zatelli

**Affiliations:** Department of Veterinary Medicine, University of Bari, 70010 Valenzano, Italy

**Keywords:** dysbiosis, dog, seizures, epileptogenesis, microbiota

## Abstract

**Simple Summary:**

Epilepsy is a common chronic neurological disease in both dogs and humans. Despite the elevated prevalence and the many advances in human and veterinary medicine, the etiology and pathophysiology of epilepsy still remain unclear. In this systematic review, the authors discussed the possible role of the gut microbiota in the canine idiopathic epilepsy etiopathogenesis via the gut–brain axis.

**Abstract:**

Canine idiopathic epilepsy is a common neurological disease characterized by the enduring predisposition of the cerebral cortex to generate seizures. An etiological explanation has not been fully identified in humans and dogs, and, among the presumed causes, several studies support the possible involvement of gut microbiota. In this review, the authors summarize the evidence of the reasonable role of gut microbiota in epilepsy through the so-called gut–brain axis. The authors provide an overview of recent clinical and preclinical studies in humans and dogs in which the modulation of intestinal permeability, the alteration of local immune response, and the alteration in production of essential metabolites and neurotransmitters associated with dysbiosis could be responsible for the pathogenesis of canine epilepsy. A systematic review of the literature, following the PRISMA guidelines, was performed in two databases (PubMed and Web of Science). Eleven studies were included and reviewed supporting the connection between gut microbiota and epilepsy via the gut–brain axis.

## 1. Introduction

**Figure 1:**
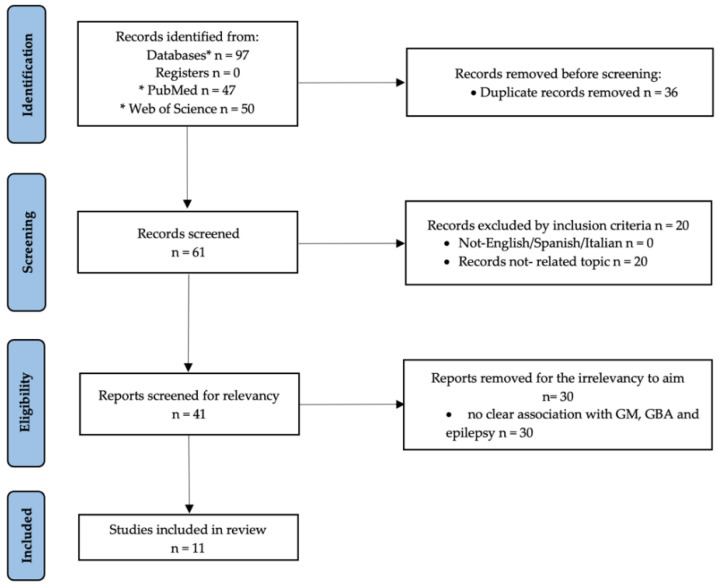
Study selection based on PRISMA flowchart.

The bidirectional communication network between the gut microbiota (GM) and brain, which includes neural, endocrine, metabolic, and immune system/pathways, is known as the gut–brain axis (GBA) [1,2,3,4]. In the last decades, it has been extensively investigated that GM can contribute to the central nervous system (CNS) development (e.g., neurogenesis, microglia maturation, and myelination) [5,6] and functions (e.g., cognition, mood, and behavior) such as sociability and anxiety [1,7]. In addition, the GM has been demonstrated to play key roles in the pathogenesis and progression of several neurodegenerative disorders (e.g., Parkinson’s and Alzheimer’s diseases, schizophrenia, autism spectrum disorder, and multiple sclerosis) [8,9,10,11,12]. Other studies have, however, also documented the effects that selected neurodegenerative diseases exert on GM composition and function via, for instance, gastroenteric inflammation and dysfunction [13]. The implication of GM in epileptogenesis has recently emerged on the basis of data collected in human patients. Indeed, environmental factors are known to exert a significant influence on GM composition which, in turn, may predispose toward the occurrence of seizures [14]. Epilepsy is a common chronic neurological disease in both dogs and humans [15,16] characterized by an enduring brain predisposition to generate unprovoked epileptic seizures >24 h apart [17]. The basic pathophysiology of epilepsy is still not fully understood even if several mechanisms have been thought responsible for seizures occurring such as abnormal discharging in specific areas of the brain because of alteration in inhibitory/excitatory balance, abnormal transmembrane ion concentrations, alteration in neuronal synaptic connectivity, alteration in the function of neurotransmitters [(such as glutamate and γ-aminobutyric acid (GABA)], and alterations in glucose and oxygen metabolism [18]. The GM, through the GBA, seems to modulate several of these pathogenetic mechanisms responsible for epileptogenesis. For this reason, in this review, the authors provide an overview of the likely role of the human and canine GM via GBA in the pathophysiology and etiopathogenesis of epilepsy and identify possible biomarkers for epileptogenesis that may reveal useful for the development of novel appropriate therapeutic approaches. A systematic literature search was conducted according to the Preferred Reporting Items for Systematic reviews and Meta-Analyses (PRISMA) 2020 statement [19]. The published research outputs on GM and epilepsy were retrieved from two databases, Pubmed/MEDLINE and Web of Science, in July 2022. The keywords used for literature searches were “gut microbiota”, “gut–brain axis”, “epilepsy”, “human” and “gut microbiota”, “gut brain axis”, ns “canine OR dog epilepsy”, using the Boolean operator “AND”. To be considered, articles were required to meet the following inclusion criteria: (i) in the Italian, English, or Spanish languages: (ii) observational studies such as cross-sectional and longitudinal studies; (iii) experimental (i.e., randomized control trials) and/or quasi-experimental studies (nonrandomized control studies); (iv) descriptive or systematic reviews and meta-analysis; (v) accessible abstract and full text. Case reports, editorials, and conference/symposium abstracts were excluded. Articles not related to the possible pathogenetic role of GM in epilepsy, or papers related to the modification of GM in epileptic humans or animals after diet or drug supplementation were ruled out. The initial literature search from both databases yielded a total of 97 records (Figure 1). After removal of duplicates (*n* = 36), 61 records were screened. Out of these 61 records, 20 of them were removed because not strictly related to the topic. Then, out of these 41 records, 30 of them were excluded due to the absence of data on the correlation among GM, GBA, and epilepsy (Figure 1). Thus, a total of 11 articles were considered eligible for this review [9,14,20,21,22,23,24,25,26,27,28] and were included (Table 1).

## 2. Gut–Brain Axis

The exact mechanisms through which the brain and gut communicate are not yet fully understood [29]; however, this crosstalk is anatomically based on the function of the autonomic nervous system (ANS), as well as through endocrine, immune, and metabolic pathways [29,30]. Anatomically, sensory information from the periphery to the CNS is transmitted via the enteric nervous system (ENS), which includes the ganglia of the myenteric plexus (or Auerbach’s), the ganglia of the submucosal plexus (or Meissner’s), and the intestinal glial cells; the prevertebral ganglia regulate the peripheral visceral reflexes while the vagus nerve, with its afferent fibers, reaches the nuclei of the brainstem. From here, afferent fibers reach the cortical and subcortical centers by crossing the thalamus and the limbic system; other fibers that are independent from the vagus nerve, i.e., the spinothalamic bundles, reach the gracilis and cuneate nuclei, from which they spread through the medial lemniscus to the thalamus and the cortex [29]. Functionally, ANS mediates the production of intestinal mucus and, thus, plays a key role in shaping the environment where the GM resides [31]. The ANS also contributes to intestinal immune activation, both directly by modulating macrophage and mast cell production and indirectly by altering the permeability of the intestinal epithelium [32,33,34,35,36]. On the other hand, key information pertaining to the intestinal lumen (such as hyperosmolarity, carbohydrate concentration, mechanical distortions of the mucosa, and presence of toxic pharmacological substances and bacterial products) reaches the CNS through signals mediated by serotonin, cholecystokinin, histamine, secretin, and melatonin, mediators released by neuroendocrine cells at the level of the intestinal mucosa [37,38,39]. In this bidirectional communication model, the GM serves as a key mediator between the enteric system and the CNS [39]. The integrity and efficiency of body barriers are also maintained by intestinal microorganisms via the activity of short-chain fatty acids (SCFAs) that induce the expression of epithelial and endothelial tight junction proteins [26]. SCFAs effectively regulate the permeability of the intestinal epithelial barrier by preventing infiltration by potentially pathogenic bacterial cells. The integrity of the blood–brain barrier (BBB) is also established by the same intercellular splicing mechanisms provided by microbial metabolism (e.g., *Clostridium*) in association with microglia cells, astrocytes, and oligodendrocytes [26,40]. Indeed, germ-free mice display increased permeability of the BBB to molecules of peripheral origin, while the administration of SCFAs followed by fecal transplantation in guinea pigs results in strengthening of tight junctions of both the BBB and the enteric barrier [41]. Conversely, alterations of the intestinal mucosal barrier observed over the course of dysbiosis and concomitant circulation of lipopolysaccharide, pathogens, cytokines, and other metabolites of microbial origin result in the activation of immune responses and subsequent onset of a local inflammatory process [29,31]. Therefore, circulating neurotoxic substances that alter the BBB via activation of the microglia may reach the brain and, thus, initiate neuroinflammatory processes [26,29]. SCFAs are also involved in controlling peripheral inflammation by the ANS; these products of dietary fiber fermentation are absorbed by the enteric mucosa and stimulate the sympathetic branch of the peripheral nervous system, which reduces local inflammatory response; it has been hypothesized that SCFAs may act on the microglia, counteracting neuroinflammation [26,27]. Increased levels of SCFAs have also been shown to stimulate, through the sympathetic afferent fibers, the release of serotonin; this, in turn, acts on the CNS, promoting learning processes and memory development [42,43]. Decreased SCFA production may, on the other hand, severely affect immune responses, cell turnover of the enteric epithelium, and several other functions of the central and peripheral nervous system. For instance, SCFAs are a source of ketone bodies that serve as ATP resources at the brain level. Administration of SCFAs also exerts beneficial effects in Alzheimer’s patients with suspected glucose dysmetabolism [27]. The metabolism of several microorganisms, including *Candida* spp., *Streptococcus* spp., and *Enterococcus* spp., as well as some sporogenous bacteria and *Escherichia coli*, results in the production of serotonin; in particular, the GM metabolizes dietary tryptophan into 5-hydroxytryptophan and, by interacting with TLR cell receptors on enterocromaffin cells (also known as enteroendocrine cells), stimulates its conversion into serotonin [4,29,40,44]. Consequently, the GM plays key roles in the serotonergic modulation of the ANS and, thus, in enteric motility, secretion, and pain perception, in addition to serotonin-regulated mood and cognitive functions at CNS level [43,45]. The precursor of serotonin, tryptophan, is a molecule with strong neurotrophic activity; excess tryptophan crosses the enteric barrier and is converted in the brain into quinolinic acid. The latter competes with NMDA receptors and exerts neurotoxic properties; by metabolizing the excess tryptophan and producing 5-hydroxytryptamine, the GM prevents the activation of microglia and, thus, the onset of inflammation in the brain [26]. Enteroendocrine cells are a type of enteric epithelial cells that secrete, upon microbial stimulation, hormones including cholecystokinin, peptide Y, and neuropeptide YY; beyond the intestinal barrier, these hormones interact with the ENS, as well as with the afferent fibers of the ANS [40,43]. In addition, the GM produces most neurotransmitters, such as GABA, serotonin and melatonin (mutual antagonists), histamine (NMDA-dependent action), acetylcholine, and catecholamines such as dopamine and norepinephrine; these are absorbed through the intestinal mucosa and exert long-range effects in numerous organs, including the brain [4,27,29,42,44]. In particular, selected *Bacillus* spp. and *Lactobacillus* spp. produce dopamine with GABAergic-like action, while other *Lactobacillus* spp. secrete acetylcholine (excitatory neurotransmitter). The genus *Lactobacillus*, along with *Bifidobacterium* spp., also produces GABA by metabolizing glutamate (excitatory); imbalances in these populations of bacteria, e.g., during dysbiosis, may result in deficiency of inhibitory neurotransmitters that, in turn, may lead to neurological disorders, including senile dementia, Alzheimer’s disease, anxiety, and depression [26,27,43]. The bidirectional communication between the GM and the brain is also supported by the hypothalamic–pituitary–adrenal axis (HPA). The HPA produces corticotrophin-releasing factor (CRF), an adrenocorticotropic hormone (ACTH) that, via subsequent release of glucocorticoids (e.g., cortisol, corticosterone, deoxy-corticosterone, and corticotrophin) and catecholamine downstream pathways, modulates the stress response [4,27,42,46]. Stressogenic stimuli determine, at the level of the hypothalamus, the release of corticotropin-releasing factor (CRF), which stimulates the pituitary gland to secrete adrenocorticotropic hormone (ACTH); ACTH reaches the adrenal glands via the bloodstream and induces the production of cortisol, the main stress hormone. The latter acts on various organs and tissues, including the digestive tract and the central and peripheral nervous system, by regulating the activity of enteric epithelial or muscular cells, enterochromaffin cells, immune cells, and the neurons of the enteric plexuses. Indeed, cortisol modulates both quality and quantity of enteric mucosal secretions, thus influencing intestinal permeability; stressful conditions induce a reduction in the expression of tight junction proteins at the level of enteric mucosa in rats and a concomitant increase in colonic permeability that, in turn, compromises immune function and GM composition [27,42]. Alterations in GM that follow cortisol-induced changes in the gut microenvironment are in turn responsible for changes in local immune responses, body metabolism, and vagal stimulation [4]. Conversely, intestinal microorganisms stimulate the production by enterochromaffin cells of galanin, a peptide that acts on the HPA by inducing the release of CRF and ACTH, which, in turn, stimulate the production of glucocorticoids by the adrenal glands; galanin also stimulates cortisol production in cortical cells and, thus, the release of norepinephrine from the adrenal medulla [42]. The GM promotes and induces the development of the enteric immune system and, via the GBA, regulates the systemic and brain immune responses [4,29,40]. By binding to Toll-like receptors (TLRs) on innate immune cells, the GM stimulates the local production of pro- and anti-inflammatory cytokines, thereby modulating the permeability of the mucosal barrier; TLRs are also expressed by enteric plexus neurons, microglia, and glial cells [29]. The circulation of pathogens and/or their metabolites or antigens in the bloodstream may induce inflammatory processes with CNS involvement and possible secondary neurological disorders [29]. The BBB and the brain lymphatic system contribute to maintenance of brain homeostasis and mechanical protection from pathogen infiltration [4]. Segmented filamentous bacteria are known to play important roles in immune response modulation. This population of bacteria adheres tenaciously to the enteric mucosa and, by interacting with submucosal cells, stimulates the maturation of B and T lymphocytes, particularly Th-1 and Th-17 cells [4,29,40]. In humans, *Bacteroides fragilis*, via exposure of polysaccharide A and binding with TLR-2, induces the maturation of regulatory T cells and the production of anti-inflammatory and neuroprotective interleukin-10 [4,29,40]. Similarly, SCFAs produced by *Clostridium* spp. promote the differentiation and activation of immunosuppressive T-reg cells, thus reducing inflammatory processes [4,29,40]. Encephalic resident immune cells (macrophages, CD8 T lymphocytes, regulatory T cells, and CD4 T-helper lymphocytes) are also modulated by innate and acquired responses elicited by the GM. In fact, intestinal activation by Th-1 and Th-17 cells can promote lymphocyte infiltration in the brain, with consequent activation of local immune cells and onset of neuroinflammation. Conversely, Th-2-mediated immune responses exert a protective effect on neuronal homeostasis via production of interleukin-4 and interleukin-10 [4,40]. B lymphocytes also contribute to neuroinflammation through the activation of the humoral immune response and production of CNS-reactive auto-antibodies, as it has been hypothesized for pediatric autoimmune neuropsychiatric disorders (PANDAS) associated with streptococcal infection [4]. The activation of the innate immune response in the brain protects the CNS from possible pathogens or infections, in addition to promoting the development, remodeling, and plasticity of neuronal circuits [26,41]. In addition to a small proportion of peripheral immune system cells, a number of innate immunity cells that originate from the yolk sac erythromyeloid line reside in the brain, i.e., astrocytes, oligodendrocytes, and microglia cells [26,41]. The latter are the most numerous and are responsible for complement fraction-mediated phagocytosis, antigen exposure and presentation via major histocompatibility complex type 1 (MHC-1), and production of pro- and anti-inflammatory cytokines [4]. Astrocytes are the most prevalent macroglia cells and perform numerous supportive biological functions, including formation and maintenance of the integrity of the BBB, regulation of ion gradient balance, neurotransmitter turnover, control of cerebral blood perfusion, and transport of nutrients to neurons [4,40]. Astrocytes also convey information from other glial cells, neurons, and immune and vascular cells to regulate neuronal excitability and the formation of new synapses. These cells are also critical to encephalic metabolism, as they hold the largest reserve of glycogen in the brain. Together with microglial cells, astrocytes also exert immune functions; indeed, these cells recognize bacterial MAMPs through the expression of membrane TLRs and process bacterial cells via major histocompatibility complex type 2 (MHC-2), thus activating the production of cytokines and modulating neuroinflammatory processes [4]. The immunomodulatory properties of macro- and microglia cells also participate in processes involving the development, homeostasis, and plasticity of cells and neuronal connections, from before birth [4,40,41]. Indeed, prior to the formation of the BBB during embryonic development, these cells migrate from the yolk sac to the brain; here, they undergo maturation while simultaneously acting on brain development [4,40,41]. Here, microglia and astrocytes produce neurotrophic and neurotoxic factors that regulate neurogenesis, differentiation or apoptosis of neurons, as well as remodel axon length and excess synaptic connections. The latter task is performed through phagocytosis activated by complement and cytokines [4,26,41]. Oligodendrocytes interact with neighboring neuronal cells through cytoplasmic branches and induce myelination of neurons that underpins the development of cognitive function [41]. In adulthood, micro- and macroglia cells only fulfil immune and homeostasis-regulating functions; indeed, the unique branching morphology of these cells allows constant surveillance of the brain environment via dynamic extension and retraction of the cytoplasmic processes that communicate with surrounding cells [4,40]. Microglial cells alternate between two physiological states, i.e., a surveillance state that underpins regulation of encephalic homeostasis, and a reactive state that activates immune responses and tissue repair. When brain tissue damage occurs, microglia cells retract their cellular processes and become amoeboid, after which they produce pro-inflammatory cytokines (interleukin-6, interleukin-12, interleukin-1β, and TNF-α) and oxidant molecules, such as reactive oxygen species and nitric oxide. Once the insult is removed, these cells release cytokines (interleukin-4, interleukin-10, and TGF-β) that inhibit inflammation event and prevent further cellular damage [26]. The maturation and development of the CNS, as well as the transition of microglia from one physiological state to another, are regulated by both intrinsic and extrinsic factors [26,40,41], which include cytokines, chemokines, and neurotransmitters, as well as gut microbial metabolites that, under physiological or pathological conditions, can cross the BBB [26,40,41]. The influence of the GM on brain development during intrauterine development is well known; indeed, maternal microbial metabolites, products of bacterial fermentations, lipopolysaccharides, and microbial peptidoglycans can cross the placental barrier [41]. Activation of the maternal immune system is also known to influence the physiological, neuropathological, and behavioral development of the unborn child [41]. Indeed, germ-free mice or antibiotic-treated animal models display neurological deficits in learning and memory, as well as altered cognitive status and emotional behaviors as adults [26,40,41]. Gut microbes are also known to modulate the myelination process and encephalic immune response via SCFAs that directly or indirectly promote microglia maturation and function; indeed, in germ-free mice, microglial cells are less responsive to viral or bacterial infections [4,41]. Astrocyte functions are also regulated by the GM. Indeed, during eubiosis, enteric microorganisms produce indole from tryptophan; the latter binds aryl hydrocarbon receptors expressed by astrocytes and stimulate anti-inflammatory activities [26]. In contrast, dysbiosis leads to a reduction in tryptophan catabolism and a subsequent lack of activation of aryl hydrocarbon receptors in the CNS, which results in increased levels of proinflammatory TNF-α, nitric oxide, and interleukin-6 [4].

## 3. Gut–Brain Axis Gut Microbiota and Epilepsy

Neuroinflammation is well known to be involved in the progression of neurodegenerative diseases, and it plays a fundamental role in the onset and the chronicization of epileptic seizures [20,21]. However, epilepsy itself also induces inflammatory responses in the brain by activating resident immune cells, i.e., macro- and microglia, and stimulating the production of proinflammatory cytokines [22]. Indeed, reactive astrocytes expressing different inflammatory mediators have been detected in the brain tissue of epilepsy patients [21]. During epilepsy, inflammatory activation of glial cells leads to the production of interleukin-1-beta, interleukin-6, and TNF-α, which are able to perpetuate inflammation to other neurons and recruit adaptive immune response cells in the brain [21]. In particular, interleukin-1-beta affects the permeability of BBB by destroying tight junctions and/or producing nitric oxide and activating matrix metalloproteases in endothelial cells [21]. Increased BBB permeability further promotes the diapedesis of adaptive immune response cells and inflammatory state progression [21]. In addition, interleukin-1-beta induces the phosphorylation of the NMDA receptor, with a subsequent increase in calcium ion flux and promoting threshold value elevation at the basis of depolarization, which results in neuronal excitability and the occurrence of epileptic seizures [21]. These cytokines may also inhibit the reuptake of glutamate (excitatory neurotransmitter) at the synaptic level together with increasing its secretion by stimulating the release of TNF-α and nitric oxide by astrocytes. Interleukin-1-beta can activate glutamatergic transmission both directly and indirectly, as well as simultaneously inhibit the inhibitory transmission mediated by GABA, thus promoting an excitable environment [21,22]. TNF-α also appears to increase the frequency of AMPA-dependent excitatory postsynaptic currents in hippocampal neurons, and it determines a reduction in the functionality of GABAergic synapses at this site [21,22]. In transgenic mice, the overexpression of TNF-⍺ and interleukin-6 promotes inflammatory responses that predispose to the occurrence epileptic seizures [21]. Indeed, anti-inflammatory drugs, including steroids, exert anticonvulsant effects that allow control of epilepsy in refractory patients [21]. However, whether glial cell activation and the ensuing inflammatory process are the cause or consequence of epileptic activation in neuronal circuits is yet to be determined [21]. Indeed, while cytokine production occurs frequently in epileptogenic foci regardless of etiology, neuroinflammation has been described in other chronic neurodegenerative diseases lacking epileptic seizures [21]. It is, thus, likely that the role of the inflammation in the pathophysiology of epilepsy depends on several factors involving the tissue environment, specific brain areas, and neuronal circuits [21]. Peripheral inflammation may be accompanied by inflammatory responses in the brain, with consequent activation of micro- and macroglia cells and the release of proinflammatory cytokines, e.g., interleukin-1-beta, interleukin-6, and TNF-α [21,22,23]. Peripheral inflammation occurs in several diseases, including inflammatory bowel disease, rheumatoid arthritis, and inflammatory liver disease, and it is often associated with behavioral alterations, cognitive and memory dysfunction, and mood and sleep alterations [23]. Peripheral inflammatory processes are moreover associated with worsening neurological and neuropsychiatric pathological conditions, including seizures, depression, Alzheimer’s disease, multiple sclerosis, Parkinson’s disease, and stroke [23]. Increased susceptibility to seizures is observed in rats following induction of inflammatory colitis; in these rats, microglia activation and TNF-α production can be detected in sections of the hippocampus. Intestinal inflammation is accompanied by increased excitability of pyramidal neurons [20]. In particular, enteric proinflammatory cytokines communicate with the innate immune system in the CNS via both the bloodstream and the afferents of the vagus nerve. The microglia cells propagate the inflammation through the CNS, amplifying the production of inflammatory mediators and neuronal excitation; BBB breakdown promotes white cell infiltration in the brain, and the resulting neuroinflammation promotes the onset of seizures [23]. Therefore, a functional link between dysbiosis and epilepsy through the GBA is highly likely [24].

In addition to proinflammatory cytokines, the immune response mediated by Th-17 lymphocytes also plays a crucial role in epileptogenic mechanisms [24]; indeed, these cells produce high levels of circulating IL-17 that stimulate the release of IL-1β, IL-6, and TNF-α (responsible for neuroinflammation), in various organs and tissues, including the brain [20,22,23]. Recent studies have detected elevated levels of interleukin-17 in the serum and CSF of epileptic patients compared to healthy controls; in addition, levels IL-17 levels are significantly and directly proportional to seizure severity and frequency [24]. Alterations in GM composition and function could lead to epilepsy via the activation and proliferation of Th-17 cells [24]. Indeed, it has recently been reported that these lymphocytes can be modulated by intestinal symbiotic bacteria belonging to, e.g., the genera *Clostridium*, *Bifidobacterium*, *Ruminococcus*, and *Bacteroides,* and the species *Escherichia coli* and *Bifidobacterium adolescentis* [24]. Th-17 activation induced by segmented filamentous bacteria is sufficient to induce extraintestinal inflammatory disease, with possible encephalic involvement and neuroinflammation [25]. However, selected intestinal bacteria produce SCFAs by promoting microglia differentiation and function, thus promoting the integrity of the BBB [25]. The metabolism of tryptophan by other intestinal microbial organisms and the binding of its derivatives to aryl hydrocarbon receptors on astrocytes are key elements for neuronal transmission and CNS development and repair, limiting any local inflammatory processes [25].

## 4. Conclusions

In this article, the authors reviewed the interaction between the GM and the brain through GBA and the likely role of the dysbiosis in epileptogenesis. According to data currently available in the literature, the role of the GM in epileptic patients is worthy of further investigation and might lead to a better understanding of the pathogenetic mechanisms of epilepsy, as well as to the identification of biomarkers associated with epileptogenesis and to the development of novel therapeutic targets based on the manipulation of the GM. Intestinal dysbiosis is known to play crucial roles in several enteric and extraenteric pathological conditions, including epilepsy, in humans and dogs. In turn, epilepsy might affect enteric homeostasis, thus contributing to further alterations of the GM [28]. The GBA underpins the communication between gut and brain via neuroanatomical pathways, as well as endocrine, immune, and metabolic systems. To date, modulation of neuroinflammation by the GM through uncontrolled activation of Th-17, production of IL-1-b, IL-6, and TNF-α, as well as a reduction of SCFAs that result in BBB alterations, are the only known causal interactions that determine the onset of epilepsy. Further investigations of the GBA are, therefore, needed to better understand the mechanisms of interaction between gut and brain that may result in increased neuronal excitability and predisposition to the onset of seizures. The likely correlation between IE and dysbiosis in dogs should prompt veterinary physicians to investigate alterations in the GM of epileptic dogs in comparison with healthy controls. This newly acquired knowledge might pave the way toward the development of novel strategies to improve clinical symptoms and lifestyle of affected patients.

## Figures and Tables

**Table 1:** List of articles eligible for the role of GM and GBA in epileptogenesis according to PRISMA 2020 statement.

Author, Year	Type of Article	Study Population
Riazi K. et al., 2008 [20]	Original article	Rats
Vezzani A. et al., 2008 [21]	Review	Humans, rats, and mice
Riazi K. et al., 2010 [22]	Review	In vitro
Riazi K. et al., 2015 [23]	Original article	Rats
Wu J. et al., 2016 [24]	Review	Humans and mice
Blander J. et al., 2017 [25]	Review	Humans and mice
Haq R.A. et al., 2018 [26]	Review	Humans and mice
Ambrosini Y. et al., 2019 [27]	Review	Humans, dogs, and mice
Dahlin M. et al., 2019 [14]	Review	Rats and mice
Iannone L.F. et al., 2019 [9]	Review	Humans
Pilla R. et al., 2020 [28]	Review	Dogs

## Data Availability

Not applicable.
